# Progress of the Anatomy and Physiology of the Nervous System during the Year 1836

**Published:** 1838-01

**Authors:** 

**Affiliations:** Berlin


### PART FIFTH.

### Medical Intelligence.

---

### PROGRESS OF THE ANATOMY AND PHYSIOLOGY OF THE NERVOUS SYSTEM DURING THE YEAR 1836.

BY PROFESSOR MÜLLER, OF BERLIN.

ENQUIRIES into the minute structure of the nerves form an unusually large proportion of the scientific communications of the past year. Besides Ehrenberg's* excellent work, we have a treatise by Valentin† full of interesting observations on the ganglia and on the terminations of the nerves. Emmert‡ has traced the distribution of the extremities of the nerves in the muscles. Gottsche§ and Treviranus¶ have examined the structure of the retina, and Treviranus that of many other parts of the nervous system. Volkmann|| and Langenbeck** have directed their attention to these and similar objects, and Kronenberg†† has investigated the structure of the plexuses. These labours, as well as those of Remak,‡‡ have for their object the rich and still unexhausted topic of the minute anatomy of the nervous fibre.

The distinction of the nerves according to their fibres becomes more and more difficult. Treviranus found the fibres, in the recent subject, in the brain as well as in the nerves, for the most part straight and uniform; Volkmann, that the varicose fibres in the nerves of sense (the optic nerve) are not constant; whilst Remak's observations prove that it is impossible to classify the nerves according to their varicose or cylindrical form, as single varicose fibres were seen more or less frequently in the most different nerves. And to denominate those fibres organic, which are to be found distributed alike in the nerves of sense and motion, is forbidden by their great changeableness and the impossibility which exists to draw exact limits between the cylindrical and varicose fibres: for one and the same fibre is often alternately cylindrical and varicose, and the nervous fibres in young animals are generally more inclined to this latter form. But even the existence of varices in nervous fibres which are not exposed to any disturbing cause is become altogether a matter of doubt. Treviranus was the first who expressed this doubt. The articulated form of the fibres of the brain is, no doubt, an observed fact; but this form is produced, according to Treviranus, by the influence of air and water; according to Valentin, by tension; and, according to E. H. Weber, by water and pressure combined. Weber, therefore, examined the nervous fibres uninfluenced by these disturbing causes, by merely moistening them with albumen. He then

* Ehrenberg in Abhandlungen der K. Akademie der Wissenschaften zu Berlin aus d. J. 1834.—Berlin, 1836, p. 605.

† Valentin über den Verlauf und die letzten enden der Nerven. Nov. act. nat. Vol. xviii. p. 1. p. 51.

‡ Emmert über die Endigungsweise der Nerven in den Muskeln.—Bern, 1836. 4.

§ Pfaff's, Mittheilungen aus dem Gebiet der Medicin, Chirurgie, und Pharmacie. Neue Folge.—Altona. Heft 3. 4. Heft 5. 6. (See Brit. and For. Med. Rev., Vol. iv. 499.)

¶ Treviranus, Beiträge sur Aufklärung des organischen Lebens.—Bremen II.

|| Volkmann, Beiträge zur Physiologie des Gesichtssinns.— Leipz 1836. 8.

** Langenbeck de Retina Observationes Anatomico-pathologicæ.—Götting., 1836. 4.

†† Plexuum Nervorum Structura et Virtutes.—Berolini, 1836. 8.

‡‡ Müll. arch. p. 145.

found, in common with Valentin, that, when he was able to isolate thin lamellæ of the brain, as, for instance, the delicate layers of the valvula cerebri, without pressure or stretching, the fibres had the appearance of nearly uniform tubes; and both observers agree in ascribing this structure to the fibres of the brain. There seems, indeed, to be no longer any doubt that the fibres of the brain and of the nerves, examined in a recent state, and independent of all disturbing causes, approach mostly to the cylindrical form, though they are not everywhere perfectly uniform, or free from slight irregularities. It is always, however, a characteristic of the fibres of the brain and of the nerves of sense, that they very readily assume this form, which is the case with no other tissue; this character, therefore, cannot be omitted from a definition of these nerves, and may be usefully applied in the discrimination of doubtful cases. It is not exactly made out on what this property discovered by Ehrenberg depends; the cause is probably to be found in the unequal cohesion of the tubes or of their contents. According to E. H. Weber's observations, unevenness and bulging out of the sheath which surrounds the soft contents, (a protrusion which occurs more on one side than on the other,) arise under the eye of the observer during protracted examination. As far as the contents of these tubes are concerned, they are, according to Valentin, perfectly clear, transparent, oily, and without any trace of globules, and the fibres, with the exception of a different degree of softness in the walls in the central parts, are throughout identical in the centre and in the periphery of the nervous system. This point, namely the proportion which the sides of the tubes bear to their contents, with the discrimination of which the modern minute anatomy of the nervous fibre began, is still one of the most difficult subjects of investigation. Valentin does not express himself everywhere with equal confidence on this question. According to him, (p. 165,) the substance of the primitive fibres is everywhere a half-fluid, slightly adhesive and transparent oily substance, which, in consequence of its refractive power, shows, when isolated, a finer internal line parallel to its superficies, without being, for this reason, divisible into a tube and its contents; when isolated it assumes either the globular form of all fluids or at least that of a flattened sphere. The contents, however, (p. 92,) may readily be pressed out of the varicose fibres, so that the mere sheath remains. At p. 70, he describes the expressed contents of the tubes of the nervous fibres as a grumous mass, consisting partly of divided and curved threads, and partly of isolated irregular globules, between which again are other globules isolated, oily, perfectly transparent, regularly or irregularly swollen, and exceeding for the most part in thickness the thread-like tissue of the brain and spinal marrow.

Whether or not the minute elementary structure of the nerves is known, must still remain a matter of doubt. These large cylinders, which are called primitive fibres, greatly exceed in strength the elements of other tissues. Schwann saw, in fibres of the thickness of the primitive fibres of the mesentery of a frog, still finer fibres running out of them. Treviranus discovered stripes running in the length of the cylindrical tubes, he even saw distinctly smaller elementary cylinders in the primitive fibres, the first having a diameter of 0,0013 millimêtres, the latter of 0,0053 in a spinal nerve of the Crucian (*Cyprinus Carassius*, Linn.) In rabbits the elementary cylinders were 0,0016 in diameter, and the stronger cylinders enclosing these, and called the primitive fibres, 0,0099. In his latest observations Remak observed the contents of the nervous tubes to be a somewhat smaller, flat, and quite solid thread, or a flat pale fillet which could be isolated from the easily wrinkled tube in large pieces by pressure. He did not succeed in detecting a more minute fibrous structure in this fillet, although, in some cases, it could be split into two or more fibres.

Much also still remains to be observed in the minute anatomy of the primitive fibres of the central parts, before general physiological conclusions can be formed. In some animals there are striking exceptions, as, for instance, in the spinal marrow of the *Cyclostomata*. The spinal marrow of the *Petromyzon* is generally known to be extensible; it can easily be torn into threads, which may be regularly pulled off in the direction of its length. In the examination of these parts I find that, in the spinal marrow of the *Petromyzon marinus*, there are different kinds of fibres, consisting of flat, thin, band-like threads, as broad as the primitive fibres of the nerves of the ox. I have never seen such bands in the spinal marrow of any other animal. These flat bands exist in all parts of the spinal marrow, their edges are parallel and without swellings of any kind, and they are pale, transparent, not divided into a tube and contents, nor can any minute structure be discovered in them: they do not join together, nor do they give off branches. Besides these, there are somewhat finer threads, of which it is difficult to say whether they also are flat; and there is moreover a peculiarly fine fibrous structure, consisting of other fibres which are greatly more minute even than those already mentioned.

Concerning the distribution of the nervous fibres in apparent anastomoses and plexuses, Kronenberg's communications are very complete. His researches give additional confirmation to the opinion that the primitive fibres of the cerebral nerves continue separate up to their ultimate distribution, that they merely change from one bundle to another, a change which takes place not only in the plexus but in every part of the nervous trunks and branches. No one has hitherto studied with so much perseverance as Kronenberg the comportment of the fibres in the several plexuses: the researches which this author has made into the minute structure of the brachial plexus of the mammalia and of the lumbar plexus of the frog form a very good foundation for physiological experiments. We shall again refer to the physiological part of these researches.

The ultimate distribution of the nerves has been illustrated by the labours of Treviranus, Gottsche, Valentin, and Emmert. Although Schwann's investigations were less favorable to a loop-like termination of the primitive fibres in corresponding ones distributed to the muscles, and Treviranus stated that he had observed a sudden termination of the primitive fibres in the muscles without minuter subdivisions, the observations of Valentin and Emmert tally with the opinions of Prevost and Dumas, who had not themselves examined the primitive fibres. The same distribution has been observed by Valentin in the iris and ciliary ligament, and even in parts exclusively sensible, as in the interior of the cochlea of birds, in the ampullæ, in the sacs of the teeth, and in the skin of the frog. Breschet saw this distribution in the cochlea and ampullæ, and in his earlier examinations of the papillæ of the skin. Burdach, junior, also hit upon the same loop-like union of two fibres, as stated in a written communication to me on the examination of the nerves in the skin of a frog, and he saw these loops formed by the fibres of different branches of the nerves. It would be very difficult at present to form any conclusion from these observations respecting further propositions in the physiology of the nerves, as every such conclusion must rest upon the hypothesis that the so-called primitive fibres are really the minutest parts of the nerves. Even if this were the case, we should still hesitate to consider the loop-like union of two primitive fibres with each other as a general fact, as regards the nerves of sensation; for this does not occur in the retina of vertebrated animals and of insects, in both of which the nervous fibres continue separate to their termination. As to the organ of hearing, I have lately convinced myself that it is just as little the case in the lamina spiralis, the most important part of the cochlea of birds. The largest portion of the nerve of the cochlea lies upon the edge of the cartilage of the cochlea and distributes its branches very regularly to its substance. A number of minute fibrillæ arise from the cartilage and pass transversely close together and in parallel lines through the greater part of the breadth of the lamina spiralis. These transparent fibrillæ are much more minute than the primitive fibres of the nerves, and end indistinctly without coalescing. In all probability they are the continuations of the primitive fibres of the nerve of the cochlea which pass through the cartilage and are here divided to the utmost degree of minuteness. The difference between these fibrillæ and the darker nervous fibres before they enter upon the cartilage may perhaps depend upon the absence of their sheaths and tubes. The lamina spiralis of the cochlea of birds, first discovered by Windischmann, is undoubtedly the most important part of the cochlea; and I cannot therefore agree with Huschke in considering the arched and wrinkled vascular membrane, falsely called the membrane of hearing (Gehörblätter) as such. Breschet also agrees with what has been stated on this subject. The nerves of the cochlea are not distributed to the lining membrane (Gewolbten Haut); the sac (Flasche), on the other hand, receives numerous nerves, but I have never succeeded in tracing the loops of the primitive fibres, and observed merely plexuses consisting of bundles of minute fibrillæ.

In reference to the present state of the physiology of the nerves, the question whether or not the fibres of the nerves of sensation terminate by loops is of no moment. The conclusions to which we have arrived depend, in no degree, upon this alternative; and, as it is certain that the truncated ends of a divided nerve are as capable of sensation as the extremities of the nerves themselves, it is also clear *à priori* that if the loop-like connexions of the extremities of the fibres of the nerves of sensation do exist, it can make no difference, as, after the removal of these loops by dividing the nerves, the result remains the same. Treviranus found a papillary termination of the nervous fibres not merely in the retina, but also a similar distribution in the auditory and olfactory nerves. In these latter nerves the papillæ are more thread-like. He examined the auditory nerve in the lamina spiralis of the cochlea of young mice, and found the bony part entirely covered by thread-like papillæ, lying close the one on the other. The cylindrical fibres of the nerve pass more disunited underneath the lining membrane of the cochlea to the membranous edge of the lamina spiralis, and after making numerous gyrations in the canals which contain them, pass through small openings and make their appearance externally as minute round globules, having a diameter of from 0,0016-0,0033 millimêtres. The cylindrical fibres of the auditory nerve itself had the same diameter. Treviranus found that, in the fox, the nerves of the semicircular canals, at their entrance into the ampullæ, expand on each side into a sheet in which the cylindrical fibres subdivide into still more minute cylinders, and again reunite to form still larger cylindrical fibres. The line of this reunion is marked by a dark ring, which consists of very minute cylinders. In a turkey-cock the extremities of the nerves had the appearance of bundles of extremely minute cylinders, and seemed to spread themselves over the internal surface of the ampullæ. In a bream, the nerves, which, before their entrance into the ampullæ, had a diameter of 0,0066 millimêtres, terminated after their entrance in cylinders of 0,0010-0,0015 millimêtres. Gottsche found the extremities of the nerves of the cochlea in hares and rabbits, and of the acoustic nerve in fish (Platessa borealis) club-like. In many fish, for instance in the sturgeon, the auditory nerve divides into threads which appear truncated; but in the plaice it forms, according to Gottsche, swellings which are twice as thick as the fibres themselves: these swellings contain in their interior a cavity, and the nervous fibres themselves exhibit a complete canal. In hares the nervous cylinders terminate upon the cochlea by egg-shaped knobs, which are distinctly raised above the internal membrane of the cochlea, like flower-buds. Treviranus found the papillæ of the olfactory nerve of mice thread-like and elongated; in the mouse they lie close to each other, but in the hedgehog they are more isolated. Their diameter is from 0,003 to 0,005 millimêtres. Upon the lining membrane of the nose of the turkey-cock, turtle, and bream, he saw merely the blunt extremity of the minute cylinder, without any projection.

Enquiries into the minute anatomy of the retina have yielded a great deal of information. B. C. R Langenbeck has been able to distinguish in the retina, 1, an external granular coat, (the cortical layer;) 2, Ehrenberg's filamentous nervous coat; and 3, a vascular coat, consisting of blood-vessels, which are united by much cellular substance into a delicate membrane. He found the fibres in the nervous coat, as in the optic nerve, varicose. Volkmann also made the same observation, but states that the varicose appearance is not constant, the swellings being at times entirely absent. These discrepancies no doubt partly depend upon the time and manner of observing. In recent eyes of mammalia and fish, I saw the fibres in the optic nerve as well as in the nervous membrane without swelling of any kind, and much more minute than they appeared when examined later. In the brain of different animals also I saw the fibres, when not submitted to pressure, very frequently at least, with almost straight outlines, and when pressed upon swollen and irregular. In the situation of the *macula flava* of the retina, Langenbeck observed that the granular layer terminated only by a sharp edge; where the retina borders upon the ciliary ligament, the granular layer of the retina also terminated, and the fibrous coat becoming much thinner formed what our author calls the ciliary part of the retina; this part lies upon the ciliary ligament, overlaps the posterior edges of the ciliary processes, is continued to their anterior edges, and terminates at the junction of the corpus ciliare with the uvea. This prolongation is said to contain small articulated tubes. Langenbeck saw the vessels of the retina in connexion with those of the ciliary ligament in the fœtus of the pig. Treviranus does not recognize any *ciliary part* of the retina. At some little distance from the rounded edge of the retina he could distinguish neither the vascular membrane nor Jacob's membrane. Near this edge, however, both its surfaces were covered with delicate membranes, of which the external was homogeneous and the internal highly vascular. Both of these membranes were prolonged over the edge of the nervous coat, they then lay close together, were formed into long folds, and extended to the ciliary ligament. The most careful examination detected no nervous matter between these membranes.*

The exact relation which the coats of the retina bore to one another had hitherto remained unknown; we therefore stated some years since that its exact structure seemed problematical. Treviranus has made a great step towards the elucidation of this subject; and his discovery, published a little before his death, was the last benefit he conferred on science. The chief part of this discovery is shortly expressed in the author's own words. The optic nerve divides into nervous cylinders, which radiate upon the external surface of the retina. Each separate cylinder, or each bundle of cylinders, deviates at a certain point from the horizontal direction, and turns towards the opposite internal surface of the retina. Soon after it has changed its direction it passes through openings in a vascular network which springs from the central vein of the optic nerve. Before it reaches the internal surface of the retina it penetrates a second vascular network derived from the last branches of the central artery. After it has passed through this last it is covered by a sheath-like continuation of the vascular membrane of the retina, and ends behind the vitreous humour in the form of a papilla. The transverse diameter of the cylinder was, in the hedge-hog 0,001 millimêtres; in the rabbit 0,0033, and in birds 0,002-0,004; in the frog the cylinders had a diameter of 0,0044 millimêtres, the papillæ of 0,0066, and the latter in the *Crucian* of 0,0039-0,0040 millimêtres. Ehrenberg recognized also in the retina of frogs and fishes staff-shaped bodies, whose connexion with the nerves seemed indistinct; he saw also club-like bodies in the Schneiderian membrane of the nose. Gottsche's observations contain many interesting details in illustration of the discovery of Treviranus. The so-called granular membrane of former writers, concerning which so many different opinions have been broached, is not found in recent eyes. If we observe a perfectly recent retina, we see, according to Gottsche, an appearance similar to a thatched roof, and small distinct nervous cylinders project out of it; if we let this remain upon the glass and moisten it from time to time, we see distinct granules; the eye of a fish which has been dead from three to four hours has the same appearance. We agree perfectly in this remark. Upon the internal surface of the retina of recently killed mammalia, frogs, or fishes, we distinctly perceive the staff-like cylinders projecting from the surface, presenting an appearance like a thatched roof. Sometimes we see these cylinders externally, as Gottsche also states. Michaelis thinks that they are merely seen through the membrane, an opinion with which I fully agree. The little cylinders are easily broken off, and swim about in a detached form; when separated they are much longer than broad: in frogs I have often seen them of unequal lengths, and some of these are slightly bent.

Volkmann and E. H. Weber have confirmed the statements of Treviranus in respect to mammalia: they saw the fibres always tortuous, but without papillæ, which indeed are not present in mammalia, but merely the staff-like bodies. The fibres of the retina were obviously free from swelling, but they saw some varicose fibres (in rabbits) which were not produced by water, for they employed the aqueous humour and avoided using pressure in their observations. The swollen fibres

* Here follows an abridgment of Gottsche's dissection of the retina given in our last number.

were most visible in the brush-like expansion of the optic nerve, and were situated by the side of and between the uniform fibres. In frogs both these observers saw all the fibres at first uniform and free from enlargements.

I make the following extract from a treatise on the structure of the retina which Dr. Michaelis has communicated to me in manuscript, and which is about to appear in the *Nov. Act. Net. Cur.*

According to Michaelis the retina extends only to the ciliary ligament, for so far only does the granular membrane and the expansion of the optic nerve proceed. He found the structure most distinct in a young owl. In this case the thick, defined, projecting border of the ciliary ligament overlaps the edge of the retina, whilst half the thickness of the retina lies above, and the other half below this border. On the other hand, in the pig, the retina, prominent and abruptly cut off, lies over the ciliary ligament which firmly attaches itself by a defined margin to the inner surface of the retina. In the calf, again, the retina becomes thin at its edge and attaches itself to the ciliary ligament by a sharp border. In man the ciliary ligament projects over the retina externally by a zigzag edge, and under this covering the retina first becomes thin.

Michaelis describes four layers in the retina: an external serous layer, a granular layer, a nervous and vascular layer, and lastly an internal serous layer. The external layer is the membrana Jacobi: in mammalia this membrane shows under the microscope only slight dimples; in the heron they appear in regular and clear divisions sprinkled with small blood-red globules, having a diameter of $\frac{1}{300}$ of a line, and between these there are still other globules more minute and of a citron-yellow colour: these globules adhere only to the external surface. In owlets they are of a bright yellow colour. The second or granular coat is the thickest membrane of the retina, and exhibits both on its external and internal surface a globular formation; when fresh it is as clear as water, but after death and under the influence of chemical agents it becomes opaque, whilst the nervous membrane remains transparent both in spirit and water. When divided, the cut surface seems to consist of cylinders standing upright, closely packed, and bearing each upon the extremity which is in contact with the nervous coat a small globule. It appears to be the substratum of the nervous threads which run upon it; in the situation of the so-called foramen centrale it becomes thin and has the appearance of a mere granular coat. The fasciculi of the nervous coat are best shewn by means of spirit of creosote. Our author states the thickness of the fibres at $\frac{1}{2500}$ of a line. These fibres have very slight and infrequent swellings; in fish they are quite uniform. The trunks of the vessels lie internal to the nervous coat. The serous or innermost coat is seen by maceration in dilute sulphuric acid. On the surface which is in contact with the retina hang a number of minute globules having a diameter of $\frac{1}{1505}$ lines, and separated from each other by tolerably regular intervals of $\frac{1}{100}$-$\frac{1}{50}$ lines. Most of these little globules are furnished with fine threads of different lengths which resemble the primitive fibres of the nerves. Michaelis considers these threads as the terminations of the nerves. The serous membrane always remains attached to the retina after the separation of the vitreous humour. Our author has not been able to ascertain the exact connexion of the tunica Jacobi and of the internal serous membrane of the retina with the ciliary ligament. The thickness of the retina gradually decreases towards its anterior edge, where the primitive fibres are separate and do not touch each other.

Michaelis has made many observations on the macula lutea; at this spot the granular membrane is very thin, but begins to increase in thickness at $\frac{1}{10}$ line from its centre, and obtains its greatest thickness at a distance of $\frac{1}{2}$ line. This strong ring or ridge around the foramen centrale, which foramen is formed by a single layer of little globules, is the macula lutea. The fibres of the retina have a peculiar arrangement around this point; for whilst, in other situations, the nerves radiate in straight lines from the optic nerves, around the macula lutea they are arranged in the form of arches, of which one part meet in the so-called foramen centrale, the next in succession converge in a regular manner from each side towards a line which stretches from the macula lutea. Between this spot and the optic nerve the nervous fibres are less numerous, more or less straight, and perfectly distinct the one from the other. The thin transparent spot, which has obtained the name of foramen centrale, is not quite round, but star-shaped; in young subjects it is elongated.

Although the modern investigations into the structure of the nerves are highly important, many things still remain to be determined. The termination of each separate fibre of the fibrous layer in a staff-like body seems still rather a postulate than an ascertained fact. For it is of the first importance to the physiology of vision to know what proportion the number and size of the primitive fibres of the optic nerve bear to the number and size of the terminations of the nerves in the nervous coat, or to the number and thickness of the staff-like bodies. If every nervous extremity corresponded to a fibre of the optic nerve, the thickness of the retina ought to progressively diminish from the point of entrance of the optic nerve to the border of the ciliary ligament, independently of the varying thickness of the other coats of the retina. The observations of Gottsche favour this supposition. It is not easy, however, to understand how so many fibres as are necessary to furnish the staff-like bodies can be compressed into the narrow compass of the optic nerve. (Compare J. Müller's Physiology, i. p. 688, and Volkmann, a. a. O, p. 104.) At all events, the fibres of the optic nerves and the nervous fibrillæ of the fibrous coat of the retina are much more minute than the strong cylinders of other nerves. As far as I have been able to compare the optic nerve of rabbits, and its minute fibrillæ distributed to the fibrous coat of the retina, with the cylindrical tubes of other nerves of the same animal, I should say that the union of many fibrillæ of the retinæ or of the optic nerve would be necessary to occupy as much space as the primitive cylinders of any other nerve; and the extraordinary minuteness of these fibrillæ clearly shows how small must be the staff-like bodies in the retina of the rabbit. Wagner also (Burdach's Physiology, v. p. 143,) found the nervous fibres of the retina of the ox very minute, $\frac{1}{800}$ - $\frac{1}{900}$ lines, whilst the cylinders of the ciliary nerve of the same animal ranged from $\frac{1}{150}$ - $\frac{1}{300}$ lines. The primitive fibres are most easily examined in the optic nerve of fish. I have never seen any fibres so minute as in the optic nerve and retina of rabbits, and in the optic nerve of the cyprinus, (probably there are varieties here.) It is difficult to understand how the fibres, pressed into the small compass of the optic nerve, should suffice for the formation of so large a surface as that of the retina, if this surface be formed, like mosaic work, by the extremities of the nerves. But if we assume that the staff-like bodies are situated in rows upon the sides of the primitive fibres, so that a great number of them are attached to one fibre, such a supposition is at variance with the theory of sensations; for the sensation of any one part does not appear to take place in the length of the fibres of other nerves, but depends upon the number of fibres, of which each has only one origin in the brain. If the sensation of different parts took place in aliquot parts of the length of the fibres, we must suppose the mind to be present in the entire body, and in every minute part of the whole length of a fibre: with such a supposition the sensations felt by those who have lost a limb by amputation are at variance. But if sensation be conveyed to the brain only from the extremities of the nerves, a fibre can refer every change which takes place in aliquot parts of its length to one spot only. For it would be absurd to suppose that the sensation of a part depends on the different distances which the nervous principle has to travel from the various points of a fibre to the brain. If, on the other hand, we consider the superior nerves of sensation as different from the other nerves, and as participating more closely in the operations of the mind, so that this is actively percipient in the extremities of the nerves of the retina, the difficulty of supposing the fibrillæ of the optic nerve sufficient to form the entire *mosaic* of the retina is at an end; but other difficulties suggest themselves, into which we cannot enquire further at present. This state of the question, which we have long considered, has determined us to mention merely those facts in the physiology of the nerves which are clearly established, conscious how many things yet remain unexplained and contradictory in the still unfinished theory of sensation.

The observations on the decussation of the optic nerves refer merely to the general course of the fibres, in respect to their crossing or not crossing. Whether there were transverse unions or divisions could be ascertained only by minute dissection. In the decussation of the optic nerves Volkmann saw no divisions of the fibres. I also looked for such divisions in vain, even when the crossing of the crossing parts was very perceptible. Treviranus says that many of the internal fibres appear to come in an arched form from both sides, and to anastomose with each other; but that these connexions, when they take place, never extend to the primitive fibres. He also saw them cross one another without uniting.

The terminations of the fibres in the brain have been examined by Valentin. The primitive fibres of the nerves which join the spinal marrow do not terminate there, but pass on to the brain. The primitive fibres which unite with the extremity of the spinal marrow run forwards; those, on the other hand, which come literally from the higher nerves go first transversely inwards to or near the cineritious substance, and then pass on in a longitudinal direction to the brain. In the true medullary substance the fibres lie side by side, but where the medullary and cineritious substances touch each other they receive between them the globules of the cineritious substance, which we shall presently mention, and radiate through the cortical substance. Here they form loop-like terminations, as in the peripheral extremities of the nerves. These fibres are most distinctly seen at the union of the medullary and cineritious substance, or in the yellow substance at the periphery of the hemispheres of the cerebrum and cerebellum. In examining the latter, this appearance has presented itself here and there. The existence of different systems of fibres on the surface of the cerebrum, those namely of the crura cerebri and commissures, and especially of the corpus callosum, and the presence of three distinct sets of fibres in the cerebellum, renders it doubtful to which system the different loops of fibres belong. These loops do not appear to me to be exactly traceable to the continued nervous fibres; and we must give up the idea of a complete and continuous circle of the nervous fibres, to which even the observations of E. H. Weber concerning the origin of the nervus trochlearis in the central line of the valvula cerebri are not favorable.

The investigations also of the ganglia, and of the grey substance of the central parts of the nervous system, have been very fruitful in results. In the interior of the ganglia of the non-vertebrated animals (leech, common snail,) Ehrenberg observed club-like bodies. In the ganglia of the leech these clubs form eight bundles, of which each pair pass through long tubes into the four roots of the ganglion. These clubs contain in their swollen parts a nucleus, and in the leech, in addition to these, numerous small globules. Valentin has described similar bodies in the abdominal ganglia of the leech. He saw globules which, like those of the ganglia of the higher animals, contained a nucleus. On a spot near the surface of this nucleus is placed a larger and redder body, and sometimes many swollen ones.—*Jahresbericht über die Fortschritte der anatomisch-physiologischen Wissenschaften im Jahre* 1836.—*Archiv. fur Anat. Jahrg.* 1837. Heft. iii.

